# Re-excision and survival following breast conserving surgery in early stage breast cancer patients: a population-based study

**DOI:** 10.1186/s12913-018-2882-7

**Published:** 2018-02-08

**Authors:** Stacey Fisher, Yutaka Yasui, Kelly Dabbs, Marcy Winget

**Affiliations:** 1 School of Public Health, University of Alberta, Edmonton, AB T6G 1C9 Canada; 2 Department of Surgery, University of Alberta, Edmonton, AB T6G 1C9 Canada; 3 Department of Medicine, School of Medicine, Stanford University, 1265 Welch Rd, Room X214, Stanford, California, 94305 USA

**Keywords:** Breast cancer surgery, Breast conserving surgery, Population-based, Re-excision, Survival

## Abstract

**Background:**

Increasing population-based evidence suggests that patients who receive breast conserving surgery (BCS) plus radiotherapy have superior survival than those who receive mastectomy. It is unclear, however, how BCS followed by re-excision is associated with all-cause and breast cancer-specific mortality, and whether the BCS survival advantage is maintained if re-excision is needed. The aim of this study was to investigate the clinical, patient, provider and geographic variation associated with receipt of re-excision surgery, and to examine the relationship between re-excision and all-cause and breast cancer-specific mortality.

**Methods:**

All women diagnosed with stage I-III breast cancer in Alberta, Canada from 2002 to 2009 were identified from the Alberta Cancer Registry, of which 11,626 were eligible for study inclusion. Type of first breast cancer surgery after diagnosis, subsequent re-excisions within 1 year, surgeon (anonymized), and hospital were obtained from provincial physician claims data. Multilevel logistic regression with surgeons and hospitals as crossed random effects was used to estimate the adjusted odds ratios of re-excision by the factors of interest. Poisson regression models were fitted to compare all-cause and breast cancer-specific mortality by surgical pattern.

**Results:**

Re-excision surgery was received by 19% (*N* = 5659) of patients who initially received BCS. The adjusted odds of re-excision varied significantly by geography of surgery, and by individual surgeon among stage I and II patients beyond the variation explained by the factors investigated (Stage I OR standard deviation (SD) = 0.43; stage II OR SD = 0.39). Patients who were treated with BCS plus re-excision surgery with either mastectomy or further BCS had similar all-cause and breast cancer-specific mortality as those treated with BCS without re-excision.

**Conclusion:**

These results suggest that breast cancer patients who are treated with BCS plus re-excision surgery by either mastectomy or further BCS have similar survival as those treated with BCS without re-excision. The significant variation in the likelihood of re-excision by geography and by individual surgeon is concerning, especially given the costs to the patient associated with additional surgery and the financial costs to the health system.

## Background

Most women diagnosed with early stage breast cancer have the option of receiving either mastectomy or breast conserving surgery (BCS) plus adjuvant radiotherapy, as randomized clinical trials have reported equivalent survival outcomes [1–3]. BCS followed by radiotherapy is generally the preferred treatment option for early stage breast cancer as BCS is less invasive, associated with less morbidity and a better cosmetic outcome than mastectomy [4]. The main disadvantage of this treatment is the risk of positive resection margins that necessitate additional surgery by either further BCS or mastectomy. Re-excision is associated with greater morbidity, patient anxiety, poorer cosmetic outcome, delayed initiation of adjuvant therapies, and increased medical cost [5, 6].

In contrast to the clinical trials reporting equivalent survival outcomes regardless of initial breast cancer surgery choice, population-based studies in the United States and Canada have recently reported poorer survival for patients who receive mastectomy compared to those who receive BCS plus radiotherapy [7–11]. Existing evidence of the relationship between re-excision and mortality is lacking and poor in quality [12, 13]. The single population-based study to investigate survival after re-excision did not include an examination of breast cancer-specific mortality and used a flawed definition of survival time that introduced immortal time bias [12]. It is therefore unclear whether the observed BCS survival advantage extends to patients who receive re-excision.

The purpose of this study was to: 1) investigate the clinical, patient, provider and geographic factors associated with receipt of re-excision surgery and, 2) examine the relationship between re-excision and all-cause and breast cancer-specific mortality.

## Methods

### Study population

The Alberta Cancer Registry was used to identify women diagnosed with stage I, II or III solid tumor breast cancer (International Classification of Diseases for Oncology [ICD-O-3] code c50 [14]) from 2002 to 2009 in Alberta, Canada who did not have another cancer diagnosis within 6 months, as this may influence treatment decisions. Patients were excluded if they: 1) did not receive breast cancer surgery, 2) had a second primary breast cancer diagnosis prior to surgery, 3) had missing or incomplete billing data, and 4) did not have at least 1 year of follow-up following initial surgery, which was necessary to ensure complete re-excision exposure ascertainment.

### Data sources and variables

Two sources of data were used, the Alberta Cancer Registry and the Alberta Health Physician claims database. The Alberta Cancer Registry is a population-based cancer surveillance system, that has been awarded the highest level of certification in all years of the study for its high level of completeness and timeliness of data collection and reporting. The Alberta Health Physician claims database collects information about all procedures performed by fee-for-service physicians for billing purposes. All surgeons performing breast cancer resections in Alberta are paid on a fee-for-service basis. All patient information was de-identified, and physical and technological safeguards were put in place to protect the confidentiality of information and the privacy of patients as per the Alberta Health Information Act.

Demographic, clinical and treatment information were obtained from the Alberta Cancer Registry including: date of and age at diagnosis; geographic region of surgery; cancer stage; tumor size; nodal status; estrogen and progesterone receptor (ER/PR) status; receipt of neo-adjuvant and adjuvant chemotherapy; receipt of hormone therapy; receipt of post-operative radiotherapy; date and cause of death. Geographic region of surgery was categorized into the five administrative health zones of Alberta. Two zones are urban and suburban in population size and density (Edmonton and Calgary) and three zones are a combination of suburban, rural and remote regions (South, Central and North). Cancer stage was determined using the American Joint Committee on Cancer (AJCC) 5th edition staging rules active for years 2002 and 2003, and the 6th edition for years 2004-2009 [15, 16]. Patients diagnosed in 2002 and 2003 who received hormone therapy were classified as ER/PR positive, while those who did not were classified as ER/PR negative, since ER/PR status was not collected by the registry in these years. Patients with missing tumor size (*N* = 194) or nodal status (*N* = 161) were randomly assigned a value proportionally based on the non-missing information. The following sensitivity analyses were run to test the assumptions: 1) patients with missing tumor size were randomly assigned to be T4 and, 2) patients with missing nodal status were assigned to be N3. Results from the sensitivity analyses did not differ from those of the primary analyses. The North American Association of Central Cancer Registries has awarded the Alberta Cancer Registry the highest level of certification in all years of the study for its high level of completeness and timeliness of data collection and reporting.

Alberta Health Physician Claims data were used to identify the first breast cancer surgery after diagnosis and subsequent re-excision procedures up to 1 year after initial surgery; date and type of surgery, surgical hospital; and anonymized physician identifier were obtained. Surgery pattern, i.e. type(s) of breast cancer surgeries received, including re-excision, was classified as BCS without re-excision, BCS plus BCS re-excision, BCS plus mastectomy re-excision and initial mastectomy. Surgeon volume was defined by the number of breast surgeries performed on patients in the study cohort in each surgeon’s highest volume year during the study period, excluding re-excision surgeries. This variable is described in detail elsewhere [17].

### Statistical analyses: Re-excision receipt

Descriptive statistics were calculated for the demographic and clinical characteristics of patients whose initial surgery was BCS by cancer stage. Multi-level mixed effects logistic regression with surgeons and hospitals as crossed random effects [18] were used to estimate odds ratios of re-excision, stratified by stage, and adjusting for age at diagnosis, geographic region of surgery, surgeon volume, year of diagnosis, tumor size and nodal status. Variation of the random effects parameters is reported as the standard deviation of .the surgeon and hospital-specific random intercepts. Crossed random effects were necessary as some surgeons operated out of multiple hospitals.

### Statistical analyses: Overall survival and breast cancer-specific mortality

Two additional exclusions were made for the investigation of surgery pattern on overall survival and breast cancer-specific mortality to facilitate the comparison of patients who received standard treatments: 1) patients who did not receive radiotherapy following BCS and 2) patients who received radiotherapy following mastectomy.

Descriptive statistics were calculated for the demographic and clinical characteristics of the study patients by surgery pattern. Overall survival and breast cancer-specific mortality of patients by surgical pattern was compared by Kaplan-Meier and cumulative incidence curves, respectively, with deaths from other causes being treated as competing risks in the cumulative incidence analysis. Patients were followed from 1 year post initial surgery until the earliest of date of death or September 30, 2011. Log-rank and Gray’s [19] test statistics were used to assess differences in overall survival and breast cancer-specific mortality, respectively. Poisson regression models were fitted to compare overall survival and breast cancer-specific mortality by surgery pattern, adjusting for geographic region of surgery, year of diagnosis, ER/PR status and hormone therapy, neo-adjuvant and adjuvant chemotherapy, tumor size, nodal status, age during follow-up and time since study start. Multi-level mixed effects models with surgeons and hospitals as crossed random effects [18] were fit; no residual variation by surgeon or hospital was found, therefore, the most parsimonious model is presented. Sensitivity analyses excluding patients with T3/T4 and N2/N3 cancers did not differ from the primary analyses. All statistical analyses were performed using SAS statistical software version 9.3 (SAS Institute Cary, NC, USA) and Stata 12.1 (Stata Corp LP, TX, USA).

## Results

**Fig. 1:**
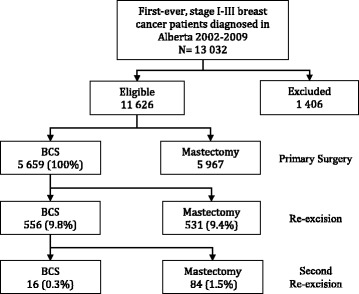
Flow chart of breast cancer surgeries received by patients in the eligible cohort

There were 11,626 eligible patients diagnosed with early stage breast cancer from 2002 to 2009 included in the study; a flow diagram of surgeries received by the eligible cohort is displayed in Fig. 1 and demographic, clinical and treatment characteristics in Table 1. Patients who did not receive breast cancer surgery (*N* = 242), had a second primary breast cancer diagnosis prior to surgery (*N* = 115), had missing or incomplete billing data (*N* = 839) or did not have at least 1 year of follow-up following initial surgery (*N* = 210) were excluded.

**Table 1:** Characteristics of breast cancer patients by surgery pattern

	Surgery Pattern			
	BCS Alone N (%)^a^	BCS, BCS N (%)^a^	BCS, Mastectomy N (%)^a^	Mastectomy N (%)^a^
Total Patients	4572 (39)	472 (4)	615 (5)	5967 (51)
Stage				
Stage I	2920 (51)	292 (5)	264 (5)	2242 (39)
Stage II	1412 (32)	161 (4)	267 (6)	2572 (58)
Stage III	240 (16)	19 (1)	84 (6)	1153 (77)
Age at Diagnosis				
< 50	1205 (39)	136 (4)	227 (7)	1541 (50)
50-59	1342 (44)	150 (5)	184 (6)	1380 (45)
60-69	1088 (42)	114 (4)	115 (4)	1304 (50)
70-79	676 (34)	60 (3)	66 (3)	1162 (59)
80+	258 (30)	12 (1)	23 (3)	580 (66)
Geography of Surgery				
Calgary	1954 (44)	175 (4)	185 (4)	2138 (48)
Edmonton	1999 (40)	206 (4)	295 (6)	2468 (50)
Central	192 (23)	23 (3)	46 (6)	563 (68)
South	308 (33)	36 (4)	56 (6)	536 (57)
North	119 (27)	32 (7)	33 (7)	262 (59)
Surgeon Volume				
Very High (60+)	2313 (44)	223 (4)	278 (5)	2485 (47)
High (20-59)	1700 (38)	172 (4)	235 (5)	2370 (53)
Medium (13-19)	362 (33)	55 (5)	67 (6)	623 (56)
Low (5-12)	148 (24)	16 (3)	28 (5)	424 (69)
Very Low (< 5)	49 (39)	6 (5)	7 (6)	65 (51)
Year of Diagnosis				
2002-2005	2083 (39)	207 (4)	296 (6)	2763 (52)
2006-2009	2489 (40)	265 (4)	319 (5)	3204 (51)
Tumor Size				
T0	2 (14)	1 (7)	0 (0)	11 (79)
T1	3523 (48)	358 (5)	373 (5)	3052 (42)
T2	997 (28)	107 (3)	200 (6)	2235 (63)
T3	34 (7)	5 (1)	38 (8)	417 (84)
T4	16 (6)	1 (0.4)	4 (1)	252 (92)
Nodal Status				
N0	3551 (47)	353 (5)	363 (5)	3324 (44)
N1	805 (28)	102 (4)	182 (6)	1789 (62)
N2	159 (21)	13 (2)	43 (6)	544 (72)
N3	57 (14)	4 (1)	27 (7)	310 (78)
ER/PR Status and Hormone Therapy				
ER/PR positive & received hormone	3035 (39)	335 (4)	431 (6)	3967 (51)
ER/PR positive & no hormone	737 (44)	57 (3)	72 (4)	807 (48)
ER/PR negative	800 (37)	80 (4)	112 (5)	1195 (55)
Neo-adjuvant Chemotherapy				
Not received	4509 (41)	454 (4)	537 (5)	5571 (50)
Received	63 (13)	18 (4)	78 (16)	403 (81)
Adjuvant Chemotherapy				
Not received	3041 (42)	307 (4)	357 (5)	3540 (49)
Received	1531 (35)	165 (4)	258 (6)	2428 (55)

*Abbreviations: BCS* breast conserving surgery, *ER* estrogen receptor, *PR* progesterone receptor

^a^Row percentages

BCS without re-excision, BCS plus BCS re-excision, BCS plus mastectomy re-excision and initial mastectomy were received by 39, 4, 5 and 51% of patients, respectively. Older patients were most likely to receive mastectomy, both initially and as a re-excision following initial BCS. BCS plus mastectomy re-excision was more prevalent than BCS plus BCS re-excision in Edmonton and in Central and Southern Alberta, while BCS plus BCS re-excision was more prevalent among patients receiving surgery in Northern Alberta, and performed in approximately equal proportions in Calgary.

### Receipt of re-excision

Patient demographic and clinical characteristics and their associations with re-excision among those who received initial BCS are shown in Table 2. Of the 5659 (49%) patients who received initial BCS, 1087 (19%) received re-excision surgery; 16, 23 and 30% of stage I, II and III cancers, respectively. Regardless of stage, the proportion of patients who received re-excision was lowest for patients 80+ years of age (9, 16 and 13% for stage I, II and III breast cancers, respectively) and when surgery was in Calgary (13, 18 and 24% for stage I, II and III breast cancers, respectively).

**Table 2:** Characteristics of stage I, II and III breast cancer patients whose initial surgery was BCS and underwent re-excision

	Stage I		Stage II		Stage III	
	Re-excision Surgery *N* (%)^a^	Total	Re-excision Surgery *N* (%)^a^	Total	Re-excision Surgery *N* (%)^a^	Total
Total Patients	556 (16)	3476	428 (23)	1840	103 (30)	343
Age at Diagnosis						
< 50	176 (21)	820	145 (23)	623	42 (34)	125
50-59	166 (16)	1031	133 (25)	535	35 (32)	110
60-69	118 (14)	862	95 (24)	391	16 (25)	64
70-79	79 (14)	582	39 (20)	194	8 (28)	29
80+	17 (9)	181	16 (16)	97	2 (13)	15
Geography of Surgery						
Calgary	184 (13)	1409	143 (18)	765	33 (24)	140
Edmonton	269 (17)	1583	188 (24)	775	44 (31)	142
Central	35 (22)	156	26 (29)	91	8 (57)	14
South	43 (18)	237	38 (29)	132	11 (35)	31
North	25 (27)	91	33 (43)	77	7 (44)	16
Surgeon Volume						
Very High (60+)	271 (15)	1761	189 (21)	888	41 (25)	165
High (20-59)	201 (16)	1265	167 (23)	723	39 (33)	119
Medium (13-19)	59 (20)	301	44 (31)	144	19 (49)	39
Low (5-12)	21 (19)	113	21 (33)	63	2 (13)	16
Very Low (< 5)	4 (10)	36	7 (32)	22	2 (50)	4
Year of Diagnosis						
2002-2005	253 (16)	1632	202 (25)	802	48 (32)	152
2006-2009	303 (16)	1844	226 (22)	1038	55 (29)	191
Tumor Size						
T0	–	–	0 (0)	1	1 (50)	2
T1	556 (16)	3476	154 (23)	660	21 (18)	118
T2	–	–	258 (22)	1147	49 (31)	157
T3	–	–	16 (50)	32	27 (60)	45
T4	–	–	–	–	5 (24)	21
Nodal Status						
N0	556 (16)	3476	158 (20)	780	2 (18)	11
N1	–	–	270 (25)	1060	14 (48)	29
N2	–	–	–	–	56 (26)	215
N3	–	–	–	–	31 (35)	88

*Abbreviations: BCS* breast conserving surgery, *ER* estrogen receptor, *PR* progesterone receptor

^a^The denominator for each percentage is the total number of patients who received initial BCS in the adjacent row for the same stage of disease

Table 3 displays the stage-specific adjusted odds ratios of re-excision. The adjusted odds of re-excision decreased with age regardless of stage. Patients that received surgery in Calgary had lower adjusted odds of re-excision than those in the other regions of the province; stage III patients treated in Central Alberta had the greatest odds of re-excision (adjusted OR = 4.13, 95% CI: 1.07, 15.9). Re-excision among stage I (OR standard deviation (SD) = 0.43, 95% CI: 0.30, 0.62) and II (OR SD = 0.39, 95% CI: 0.25, 0.61) patients varied significantly by individual surgeon, beyond the variation explained by the factors investigated.

**Table 3:** Adjusted^a^ odds ratio of re-excision estimates^b^ for stage I, II and III breast cancer patients whose initial surgery was BCS

	Adjusted^1^ Odds Ratios of Re-excision (95% CI)		
	Stage I	Stage II	Stage III
Total Patients	3476	1840	343
Age at Diagnosis	*P* < 0.001	*P* = 0.43	*P* = 0.32
< 50	1.00	1.00	1.00
50-59	0.68 (0.54, 0.87)	1.07 (0.80, 1.41)	1.06 (0.57, 1.97)
60-69	0.58 (0.44, 0.75)	1.09 (0.80, 1.49)	0.84 (0.39, 1.80)
70-79	0.56 (0.42, 0.76)	0.85 (0.56, 1.29)	0.68 (0.24, 1.90)
80+	0.35 (0.21, 0.60)	0.67 (0.37, 1.20)	0.21 (0.04, 1.22)
Geography of Surgery	*P* = 0.004	*P* = 0.04	*P* = 0.09
Calgary	1.00	1.00	1.00
Edmonton	1.74 (1.18, 2.57)	1.64 (1.09, 2.45)	2.14 (1.07, 4.29)
Central	2.26 (1.27, 4.03)	1.62 (0.86, 3.05)	4.13 (1.07, 15.9)
South	1.74 (1.00, 3.04)	1.55 (0.87, 2.75)	2.13 (0.77, 5.89)
North	2.89 (1.47, 5.68)	2.58 (1.34, 4.97)	3.79 (0.94, 15.3)
Surgeon Volume	*P* = 0.80	*P* = 0.95	*P* = 0.16
Very High (60+)	1.00	1.00	1.00
High (20-59)	0.96 (0.63, 1.45)	0.99 (0.65, 1.51)	1.40 (0.69, 2.83)
Medium (13-19)	0.96 (0.58, 1.60)	1.16 (0.67, 2.00)	1.88 (0.71, 5.01)
Low (5-12)	0.83 (0.42, 1.66)	1.22 (0.59, 2.50)	0.24 (0.04, 1.55)
Very Low (< 5)	0.51 (0.16, 1.62)	1.26 (0.45, 3.54)	1.62 (0.18, 14.2)
Year of Diagnosis	*P* = 0.42	*P* = 0.18	*P* = 0.27
2002-2005	1.00	1.00	1.00
2006-2009	1.08 (0.89, 1.32)	0.85 (0.67, 1.07)	0.74 (0.43, 1.26)
Tumor Size		*P* < 0.001	*P* < 0.001
T0/T1	1.00	1.00	1.00
T2	–	1.39 (1.04, 1.86)	1.40 (0.15, 12.8)
T3	–	6.33 (2.82, 14.2)	1.33 (0.12, 14.2)
T4	–	–	1.83 (0.17, 19.9)
Nodal Status		*P* < 0.001	*P* = 0.75
N0	1.00	1.00	1.00
N1	–	1.74 (1.30, 2.33)	1.40 (0.15, 12.8)
N2	–	–	1.33 (0.12, 14.2)
N3	–	–	1.83 (0.17, 19.9)
RE Parameter Estimates (SDs)			
Hospital	0 (−)	0 (−)	0.15 (0.00, 9.09)
Surgeon	0.43 (0.30, 0.62)	0.39 (0.25, 0.61)	0 (−)

*Abbreviations: BCS* breast conserving surgery, *CI* confidence interval, *RE* random effects, *SD* standard deviation

^a^Adjusted for the variables in the table

^b^Multi-level logistic regression with hospitals and surgeons as crossed random effects

### Overall survival and breast cancer-specific mortality

Mortality analysis includes 9023 (78%) patients: 517 patients who did not receive radiotherapy after BCS and 2086 patients who received radiotherapy after mastectomy were excluded. Median follow-up time was 4.0 years. A total of 880 (9.8%) patients died during the follow-up period: 406 (4.5%) from breast cancer and 474 (5.3%) from other causes.

**Fig. 2:**
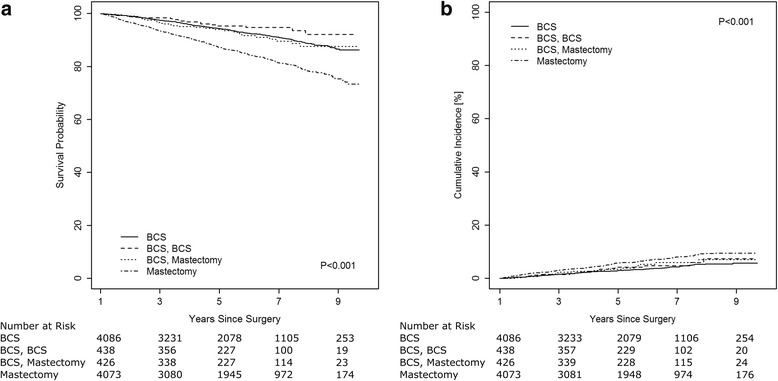
Kaplan-Meier survival probabilities (**a**) and cumulative breast cancer mortality (**b**) by surgery pattern for stage I-III breast cancer patients

Figure 2 shows Kaplan-Meier and cumulative incidence curves for overall survival and breast cancer-specific mortality, respectively, by surgery pattern. Patients who received initial mastectomy had the greatest risk of all-cause and breast cancer-specific mortality. The five-year all-cause survival probabilities for patients who received BCS without re-excision, BCS plus BCS re-excision, BCS plus mastectomy re-excision and initial mastectomy were 92.5% (95% CI: 91.5%, 93.5%), 94.7% (95% CI: 91.5%, 96.8%), 91.2% (95% CI: 87.8%, 94.3%) and 84.4% (95% CI: 83.0%, 85.7%), respectively. The five-year cumulative incidence of breast cancer death was 3.5% (95% CI: 2.9%, 4.2%), 4.7% (95% CI: 2.7%, 7.6%), 5.3% (95%: 3.1%, 11.1%) and 6.8% (95% CI: 5.9%, 7.8%) for patients who received BCS without re-excision, BCS plus BCS re-excision, BCS plus mastectomy re-excision and initial mastectomy, respectively.

Adjusted all-cause and breast cancer-specific mortality rate ratios are shown in Table 4. BCS plus BCS re-excision and BCS plus mastectomy re-excision were both associated with similar all-cause and breast cancer-specific mortality as BCS without re-excision. Patients who received initial mastectomy had significantly greater all-cause (HR = 1.35, 95% CI: 1.15, 1.58) and breast cancer-specific (HR = 1.36, 95% CI: 1.08, 1.72) mortality than those who received BCS without re-excision, although confidence intervals were wide for breast cancer-specific mortality. Marginally significantly larger all-cause mortality rate was found in Edmonton (HR = 1.17, 95% CI: 1.01, 1.37) and Southern Alberta (HR = 1.29, 95% CI: 1.02, 1.61), and significantly larger breast cancer-specific mortality rates were found in Southern (HR = 1.39, 95% CI: 1.00, 1.93) and Northern Alberta (HR = 1.62, 95% CI: 1.05, 1.93), compared to Calgary. The adjusted all-cause mortality rate decreased from 2002 to 2005 to 2006-2009 (HR = 0.83, 95% CI: 0.71, 0.98).

**Table 4:** Adjusted^a^ Poisson regression models assessing all-cause and breast cancer-specific mortality by surgery pattern for stage I-III breast cancer patients

	Adjusted^a^ Mortality Rate Ratio (95% CI)	
	All-Cause	Breast Cancer-Specific
Surgery Pattern	*P* < 0.001	*P* = 0.07
BCS	1.00	1.00
BCS, BCS	0.68 (0.43, 1.09)	1.19 (0.71, 1.97)
BCS, Mastectomy	0.93 (0.64, 1.36)	1.22 (0.74, 2.00)
Mastectomy	1.35 (1.15, 1.58)	1.36 (1.08, 1.72)
Geography of Surgery	*P* = 0.11	*P* = 0.14
Calgary	1.00	1.00
Edmonton	1.17 (1.01, 1.37)	1.13 (0.90, 1.42)
Central	1.16 (0.89, 1.50)	1.10 (0.75, 1.61)
South	1.29 (1.02, 1.61)	1.39 (1.00, 1.93)
North	1.33 (0.96, 1.85)	1.62 (1.05, 2.48)
Year of Diagnosis	*P* = 0.024	*P* = 0.89
2002 – 2005	1.00	1.00
2006 – 2009	0.83 (0.71, 0.98)	0.98 (0.78, 1.24)
ER/PR Status & Hormone therapy	*P* < 0.001	*P* < 0.001
ER/PR positive & received hormone	1.00	1.00
ER/PR positive & no hormone	1.51 (1.24, 1.84)	1.25 (0.88, 1.75)
ER/PR negative	1.96 (1.68, 2.30)	2.61 (2.10, 3.25)
Neo-adjuvant Chemotherapy	*P* = 0.11	*P* = 0.030
Not received	1.00	1.00
Received	0.73 (0.50, 1.06)	0.60 (0.39, 0.94)
Adjuvant Chemotherapy	*P* = 0.002	*P* = 0.46
Not received	1.00	1.00
Received	1.38 (1.12, 1.70)	1.11 (0.84, 1.47)
Tumor Size	*P* < 0.001	*P* < 0.001
T0/T1	1.00	1.00
T2	2.12 (1.82, 2.47)	2.36 (1.85, 3.00)
T3/T4	2.97 (2.20, 4.01)	4.34 (2.95, 6.36)
Nodal Status	*P* < 0.001	*P* < 0.001
N0	1.00	1.00
N1	1.80 (1.52, 2.13)	2.35 (1.85, 3.00)
N2/N3	3.00 (2.34, 3.86)	4.53 (3.27, 6.26)

*Abbreviations: BCS* breast conserving surgery, *CI* confidence interval, *ER* estrogen receptor, *PR* progesterone receptor

^a^Adjusted for all variables shown in the table, in addition to age during follow-up and time since study start

## Discussion

In this study, breast cancer patients who were treated with BCS plus re-excision surgery had similar all-cause and breast cancer-specific mortality as those treated with BCS without re-excision. These results supplement increasing observational evidence that in the population-based context, patients who receive mastectomy have poorer survival than those who receive BCS plus radiotherapy [7–11], suggesting that this survival advantage may extend to patients who receive re-excision surgery by either mastectomy or further BCS. The limited literature that has investigated survival of patients treated with BCS with and without re-excision has also reported comparable overall [12, 13, 20] and breast cancer-specific [12] survival, however there has been little comparison with initial mastectomy. A large population-based cohort in Denmark found comparable overall survival rates in multivariate analysis among patients treated with BCS with or without re-excision, or initial mastectomy [20]. The association of re-excision and loco-regional recurrence has been investigated more thoroughly, with inconsistent results; re-excision surgery has been associated with increased recurrence risk [13, 21, 22], while others have reported no association [23, 24]. Increased recurrence is likely due to residual confounding, but further investigation is necessary to ensure that re-excision is not being performed unnecessarily. Our results suggest that in the population context, re-excision by either BCS or mastectomy does not translate into a measurable survival discrepancy.

The percentage of patients that received re-excision in this study, 19%, is similar to that found by other population-based studies in England, Ireland and the Netherlands [12, 25, 26]. In Canada, however, re-excision rates have been reported to vary from 17% in Manitoba and Quebec to 56% in Newfoundland [27].

Re-excision use varied significantly by geography and surgeon among stage I and II patients in the current study. We believe that this is due to the lack of a formal or informal consensus regarding the best definition of negative surgical margins that adequately reduce the risk of recurrence following BCS at the time of this study [28]. A survey of Canadian surgeons in 2012 found that 40% considered a margin negative when ‘no tumor cells are seen on the inked margin’, while 14, 29 and 18% required 1 mm, 2 mm and 5 mm of clear tissue, respectively [29]. Since this study, a consensus guideline for stage I and II breast cancer patients who receive BCS followed with whole breast irradiation has been developed, concluding that margins wider than ‘no ink on tumor’ do not further reduce recurrence risk [30]. In the current study, patients who received surgery in the urban region of Calgary were consistently the least likely to receive re-excision; empirical evidence suggests this is due to early encouragement to accept ‘no ink on tumor’ margins. Regional and surgeon-specific variation may also be explained in part by variation in the use of intraoperative techniques of margin assessment including imaging, routine cavity shaving and frozen section analysis, variation of which has been reported in Canada [29]. Although it is encouraging that no residual variation by surgeon or hospital was found for all-cause or breast cancer-specific mortality, the reported geographic variation of all-cause and breast cancer-specific survival is of concern and deserves further investigation.

Surprisingly, re-excision was not associated with surgeon volume, as other studies have reported this association [26], however, other characteristics of the operating surgeon such as years since graduation, foreign training and specialization may be responsible for some of the residual variation by surgeon reported. Residual confounding by unmeasured patient case-mix variables may also explain some of the reported variation by surgeon.

Re-excision was also associated with tumor size and, among patients with stage I disease, younger age at diagnosis. BCS performed on a large tumor is a more technically challenging procedure than that performed on a small tumor as it is more difficult to remove a large amount of tissue and simultaneously optimize cosmetic outcome. Additionally, younger women tend to have dense breasts, limiting the ability of the surgeon to accurately assess tumor extent preoperatively [24]. It has also been suggested that younger patients value a satisfactory cosmetic result more than older patients and, therefore, the initial excision may be inappropriately minimized, thus requiring subsequent surgery [31].

The population perspective of the present study is especially valuable as surgical decisions for breast cancer are based on various factors including patient preference and contraindications to radiotherapy. It is therefore of clinical interest to investigate surgical patterns in a population-based and unselected series of patients with minimal bias caused by variation in access to care often present in alternatively-funded health care systems. Multi-level modeling was used to account for the hierarchical data structure and provide estimates of the level of variation within surgeons and hospitals. Limitations of the current study are largely due to the nature of the administrative datasets; information about margin status, breast density, recurrence and prognostic factors such as comorbidities, lifestyle factors or socioeconomic status were not available. Lack of these variables may introduce confounding by indication if higher risk patients are more likely to receive mastectomy. Additionally, longer follow-up may be necessary to detect survival differences between the surgery pattern groups.

## Conclusion

The current results suggest that patients who receive BCS plus re-excision with either BCS or mastectomy have similar survival outcomes as patients who receive BCS without re-excision. Although not possible in the current study, investigation of recurrence rates and surgical patterns, would be of interest. Given the increasing population-based results favoring initial treatment with BCS plus radiotherapy over mastectomy, and considering that mastectomy is a more invasive procedure, fear of additional surgery should not be a reason to receive initial mastectomy. We suggest greater efforts towards educating and encouraging women to receive initial BCS rather than mastectomy when appropriate. This may be particularly important in the face of increasing mastectomy rates [32, 33], including double mastectomy among younger women [34], many of which are likely excellent candidates for BCS. Further work should explore how factors such as possibility of re-excision with BCS and avoidance of radiotherapy with mastectomy influence surgical decision making.

Additionally, although the survival of patients who received re-excision was not significantly different from those who received a single BCS procedure, the significant variation in the likelihood of re-excision by geography and by individual surgeon is concerning, especially given the costs to the patient associated with additional surgery and the financial costs to the health system. We suggest targeted education efforts for surgeons on the recent consensus guidelines to facilitate increasing surgeon uptake, which may help to reduce variation and prevent unnecessary re-excision. Re-evaluation of re-excision rates in Alberta in the coming years will be of interest.

## Abbreviations

BCS: Breast conserving surgery
ER/PR: Estrogen and progesterone receptor
ICD-O: International Classification of Diseases for Oncology
OR: Odds ratio
SD: Standard deviation
